# Idleness

**Published:** 1879-05

**Authors:** 


IDLENESS.—No man was made to be a loafer; it is a crime against oneself, a crime against society, a crime against the Merciful One who has enacted the universal law, "In the sweat of thy face thou shalt eat bread," with the added injunction, "Be diligent in business." In this connection Mrs. Dall has truthfully and forcibly written:

"There is no greater enemy to body and soul than idleness. unless it is that public sentiment which compels to idleness. Thousands, and tens of thousands, have fallen victims to it. The woman who will not labor, rich or honored though she be, bends her head to the inevitable curse of heaven. This curse works in failing health, fading beauty, broken temper, and weary days Let her never fancy that, being neither wife nor mother, she is exempt from the law. She cannot balance that decree of God by the foolish customs of society, or the weak objections of kindred. Disease, depression, moral idiotcy or inertia, follow an idle life. He who never rests has made woman in his image; and health, beauty, force and influence. follow in the steps of labor alone."

